# Quantitative evaluation of apparent diffusion coefficient in a large multi-unit institution

**DOI:** 10.1016/j.phro.2025.100856

**Published:** 2025-10-30

**Authors:** Joshua P. Yung, Yao Ding, Ken-Pin Hwang, Carlos E. Cardenas, H.Asher Ai, Lucas McCullum, Natalie A. West, Clifton D. Fuller, R. Jason Stafford

**Affiliations:** a Department of Imaging Physics, The University of Texas MD Anderson Cancer Center, Houston, TX 77030, USA; b Department of Radiation Physics, The University of Texas MD Anderson Cancer Center, Houston, TX 77030, USA; c The University of Texas Graduate School of Biomedical Sciences, Houston, TX 77030, USA; d Department of Radiation Oncology, The University of Texas MD Anderson Cancer Center, Houston, TX 77030, USA; e UT MD Anderson Cancer Center UTHealth Houston Graduate School of Biomedical Sciences, Houston, TX 77030, USA

**Keywords:** Magnetic resonance imaging, Quality control, Diffusion weighted imaging, Quantitative

## Abstract

**Background and purpose:**

Diffusion weighted imaging (DWI) and their calculated apparent diffusion coefficient (ADC) values in magnetic resonance imaging (MRI) has grown with applications across multiple anatomical sites and settings across diagnostic and oncologic therapy settings, necessitating characterizing its quantitative performance across multiple scanners. Therefore, the purpose of this study was to determine the quantitative accuracy of DWI and their calculated ADC values across a fleet of MRI systems using a NIST-traceable diffusion phantom.

**Materials and methods:**

Three NIST/QIBA DWI phantoms were imaged with protocols provided with the phantom on a multi-vendor fleet of 23 clinical MRI scanners. User-directed regions-of-interest on each vial provided ADC measurements among a wide range of NIST-traceable ADC values. Across all the MRI systems and data, the coefficient-of-variation was calculated, and Bland-Altman analysis was performed. The measurements between phantoms were analyzed to assess their agreement to determine if multiple phantoms can be used for quality assurance across a large institution.

**Results:**

Lower ADC values and the sagittal orientation exhibited the largest error range and coefficient-of-variation (CoV) and was directly related to SNR. From a one-way analysis of the variance (ANOVA), the mean and standard deviation of the percent errors from each phantom were not significantly different from one another. No strong differences in Bland-Altman plots were seen between 1.5T and 3T scanners, however confidence intervals were different among vendors. The ADC values among the three phantoms were not significantly different from one another.

**Conclusions:**

A comprehensive analysis of institution-wide MRI scanners was conducted, motivating further work to utilize these results to implement a comprehensive quality assurance program and advanced scheduling system to better match similarly performing MRI scanners.

## Highlights

• An established diffusion phantom was scanned across 23 magnetic resonance imaging scanners, two manufacturers, and two field strengths, and consistently showed increased error and lower signal–noise-ratio at lower apparent diffusion coefficient (ADC) values.
• ADC measurements scanned in sagittal orientation consistently showed higher coefficient-of-variation across the reference ADC values.
• No significant differences were seen in Bland-Altman analysis across field strengths.

## Graphical abstract

**Figure:**
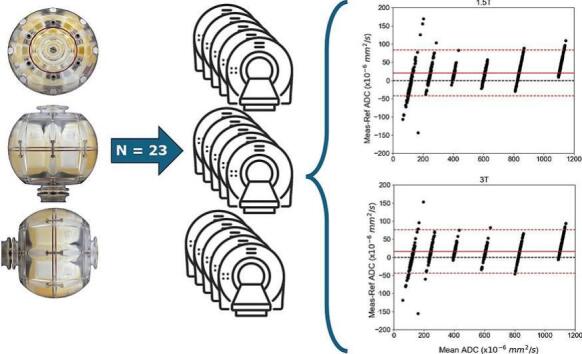

## 1 Introduction

Diffusion weighted imaging (DWI) is increasingly being used in oncologic applications for assessment of disease response and prediction of therapeutic effects [[1], [2], [3], [4], [5], [6], [7], [8], [9]]. In head and neck cancers, in particular, there is a growing body of literature, which supports, at least in preliminary institutional datasets, the utility of DWI for prognostication of radiotherapeutic or chemoradiotherapeutic response [[10], [11], [12], [13]]. DWI presents a unique advantage in radiation oncology as its ability to obtain functional information can be used for tumor delineation and to assess post-treatment changes [[14], [15], [16]]. Specifically, the apparent diffusion coefficient (ADC), a diffusion parameter derived from DWI, can be used as a quantitative imaging biomarker for disease response and toxicity effects [17].

One hurdle to the prominent implementation of ADC to the clinical workflow is the concern of repeatability on the same scanner and reproducibility across different scanners which may affect response assessment. To characterize this, one prior study has investigated ADC measurements across six MRI scanners across four institutions [18], however only one manufacturer was investigated. Another study investigated the use of an ice-water-based DWI phantom across a fleet of 35 MRI systems of three manufacturers and 1.5T and 3T field strengths [19]. A smaller study used a similar phantom across three Siemens scanners [20]. A recent development for more accurate ADC measurement validation is the National Institutes of Standards and Technology (NIST)-traceable Quantitative Imaging Biomarker Alliance (QIBA) diffusion phantom [21] from CaliberMRI (Boulder, Colorado, USA) which has been investigated in one study across six 3T scanners [22] and in another across one 1.5T scanner and three 3T scanners [23]. The largest of these studies investigated the CaliberMRI phantom across 12 scanners split evenly between 1.5T and 3T from the three major MRI manufacturers (i.e., Siemens, GE, and Philips) [24]. Although these studies assist in characterizing the behavior of ADC across multiple devices, manufacturers, field strengths, and acquisition parameters, it is necessary to continue these efforts especially in larger centers to ensure transferability and scalability of large-scale clinical trials and datasets.

Therefore, in our analysis, we aim to assess the harmonization and standardization of ADC as a quantitative imaging biomarker across a fleet of 23 MRI scanners from both Siemens and GE at both 1.5T and 3T field strengths. We also aim to utilize this detailed and comprehensive information to categorize our scanners by vendor, platform, or field strength by those which are closest in terms of ADC accuracy, so that, if necessary, patients can be selectively triaged to scanners which most closely approximate the machine of their initial imaging. For example, if the scanner of baseline MRI measurements exhibits underestimated ADC, then the scanner at follow-up should exhibit a similar bias, assuming the original scanner is unavailable, to minimize reproducibility errors. Taking this a step further, automated algorithms could be developed to determine these decisions quantitatively at an operations research level. Therefore, our study aims to provide this initial benchmarking across scanners using the QIBA consensus protocol as a precursor to longitudinal assessment amongst scanners for quality assurance as well as later lending to the investigation of variance introduced by specific clinical protocols and advanced acquisition techniques (e.g., multi-shot, simultaneous multi-slice encoding, non-Cartesian, etc.).

## 2 Materials and methods

In this study, we aimed to characterize our entire fleet of NIST/QIBA phantoms (n = 3) and MRI scanners (n = 23) which constituted nine unique platforms (Supplementary Table S1) with a total of four 1.5T platforms and four 3T platforms with four Siemens (Siemens Healthcare; Erlangen, Germany) and five GE (GE Medical Systems; Milwaukee, WI, USA) manufacturers. Note the 3T GE Signa HDxt systems had Twin Resonance Module (TRM) gradients which allowed the acquisitions to be performed in two separate performance modes (Whole and Zoom).

The NIST-traceable QIBA diffusion phantom [21] is 194 mm in diameter and holds 30 mL vials of polymer polyvinylpyrrolidone (PVP) in aqueous solution. Increasing concentrations of the polymer cause the ADC values to be reduced. The 13 vials within the phantom consist of three vials of pure water and two vials each of a PVP solution at 10, 20, 30, 40, 50 % PVP by mass fraction as shown in Supplementary Fig. S1. To eliminate the temperature sensitivity of diffusion, the phantom was filled with ice water prior to scanning and equilibrated at 0 °C according to the provided phantom instructions [21]. The temperature of the phantom was measured and verified to be within 0 ± 0.2 °C. The value of the diffusion at 0 °C as a function of PVP is given in the table below (Supplementary Table S2).

The diffusion weighted acquisition parameters followed the recommended QIBA protocol but had some platform dependent parameters [25]. The phantom was imaged using each system’s head phased-array radiofrequency receive coil which ranged from 8 to 64 channels. Imaging was performed using diffusion-weighted, single-shot echo planar imaging (EPI) acquisitions (echo time, TE = 71.8–113 ms; repetition time, TR = 10 or 15 s; field-of-view = 216 – 220 mm; matrix = 128 x 128 or 192 x 192; slice thickness = 4 mm; number of averages = 1) with four b-values (0, 500, 900, 2000 s/mm^2^) in simultaneous three orthogonal directions. If desired, the user may include more averages for each of the b-value images, typically including more averages with higher b-values due to the reduced SNR. Images were acquired single-slice and the NIST/QIBA phantoms were re-oriented for each slice orientation (axial, coronal, sagittal) to explore potential eddy current effects [26]. All of the scanners used in this study (across both GE and Siemens systems) included built-in 3D distortion correction methods which was utilized. The NIST/QIBA phantoms were first characterized to establish phantom equivalence via analysis of their variance. Subsequently, each scanner was evaluated. Two scanners were equipped with dual gradient modes and were evaluated in each mode. In total, after some scanners were scanned multiple times across the three phantoms to validate their equivalence, a total of 31 sets of data were collected.

The resulting images were analyzed using the QIBAPhan software [27] that generated user-directed regions-of-interest (ROI) and corresponding ADC maps from the four b-values and a mono-exponential curve fitting along with automated signal-to-noise ratio (SNR) measurements (Supplementary Fig. S1). Equal area ROIs of 1–2 cm diameter, following QIBA guidelines [28], were used. The quantitative measurements were compared to the NIST-measured ADC values provided by the phantom manufacturer. The coefficient of variation (CoV) was calculated as the standard deviation divided by the mean for each PVP concentration and slice orientation using the combined voxel values within each vial’s ROI. The number of voxels within each vial’s ROI varied from between 60 and 120 depending on the orientation and the exact acquisition parameters. A combined 3D CoV was also calculated using a square root sum-of-squares approach across each orientation. The dependency of the ADC accuracy to the SNR of the image was also examined. Bland-Altman plots and their respective limits of agreement [29,30] were used to investigate the ADC accuracy and bias between devices, manufacturers, and magnetic field strengths.

## 3 Results

**Fig. 1:**
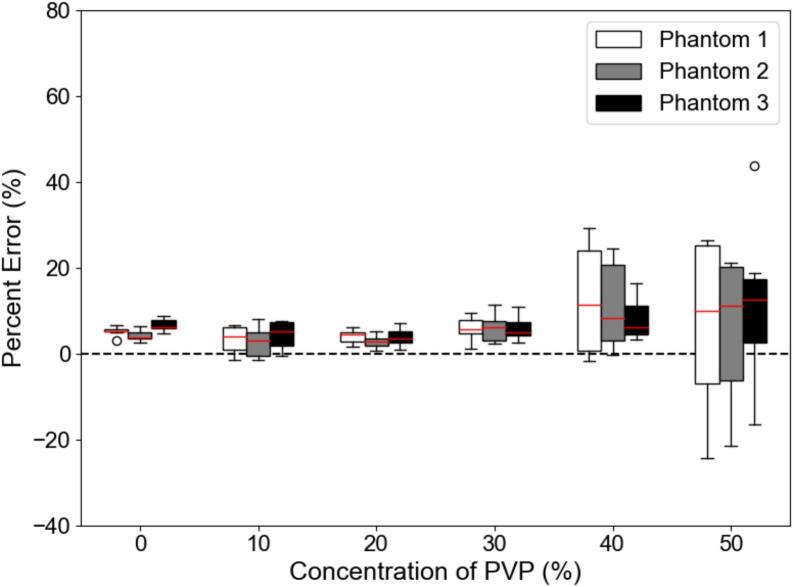
The percent error for each concentration of PVP in one MRI system that scanned all three diffusion phantoms. The median value is shown as a red horizontal line for each phantom and PVP concentration. (For interpretation of the references to colour in this figure legend, the reader is referred to the web version of this article.)

**Fig. 2:**
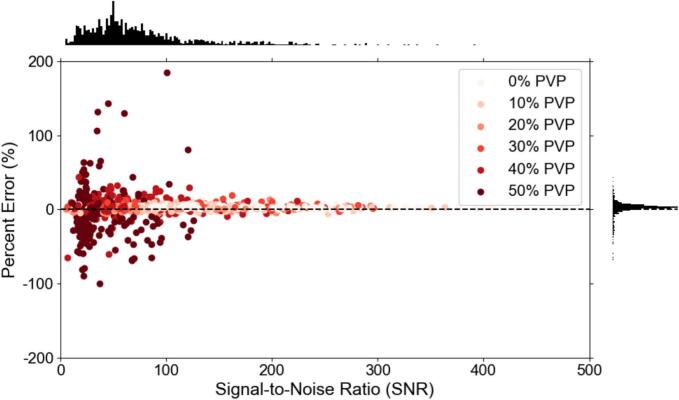
Scatter plot of the software-measured signal-to-noise ratio versus the percent error in the measurement. Accompanying per-axis histograms are shown on the top and right side to better visualize the average SNR (top) and percent error (right). The PVP concentrations are shown in varying shades of red to better analyze their trends. (For interpretation of the references to colour in this figure legend, the reader is referred to the web version of this article.)

In Fig. 1, a comparison of the three diffusion phantoms used in this study is shown. Each phantom was scanned on the same MRI system and the percent error of the ADC measurement compared to the known value was calculated. The percent error was consistently less than 10 % except for the 50 % PVP concentration which corresponded to the lowest ADC value (118.3–123.0 x10^-6^ mm^2^/s). Standard deviation also increased predictably as the ADC values decreased. In the one-way analysis of the variance (ANOVA) the ADC values between the diffusion phantoms were not statistically significant (10 % PVP: F = 0.34, p = 0.72, 20 % PVP: F = 0.88, p = 0.44, 30 % PVP: F = 0.01, p = 0.99, 40 % PVP: F = 0.31, p = 0.74, 50 % PVP: F = 0.22, p = 0.80) except for the water references (0 % PVP: F = 10.3, p < 0.05), where the errors were the smallest. The large increase in error and standard deviation appears to be directly related to the SNR (Fig. 2).

**Fig. 3:**
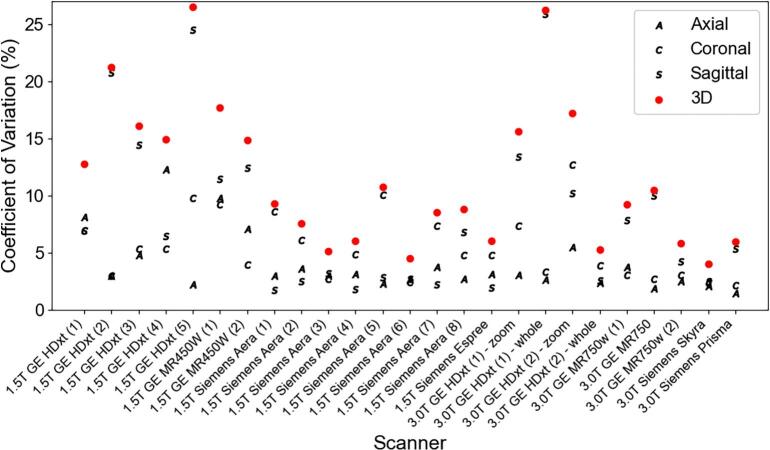
Plot of the average CoV across all PVP concentrations for each MRI system among the different slice orientations (black letters) and the combined 3D CoV (red circle). The numbers in parentheses for the x-axis labels differentiate individual scanners of the same vendor and system. (For interpretation of the references to colour in this figure legend, the reader is referred to the web version of this article.)

The CoV was calculated using all the ADC voxel values inside the ROIs for each dataset with respect to the axial, coronal, and sagittal slice orientation (Fig. 3). No measured noise is assumed to have a CoV of 0 % while a CoV of 100 % indicated that the standard deviation of the ADC values within the vial equal the mean ADC of that vial. For most scanners, the sagittal slice orientation had the highest CoV among the different orientations.

**Fig. 4:**
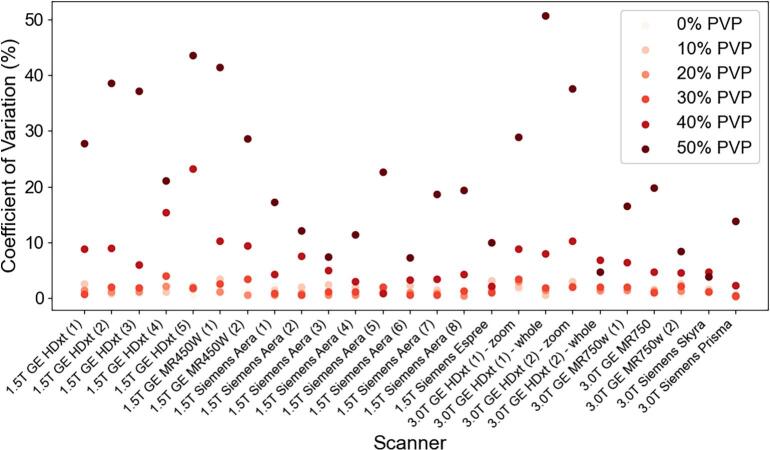
Plot of the average coefficient of variation across all slice orientations for each MRI system among the different vial PVP concentrations. The numbers in parentheses for the x-axis labels differentiate individual scanners of the same vendor and system.

Additionally, the CoV was calculated using all the ADC voxel values inside the ROIs for each dataset with respect to the PVP concentration of each vial (Fig. 4). Across all the MRI systems and datasets, the vials with the higher PVP concentrations had the higher CoV. These vials also corresponded to the lowest SNR from Fig. 2.

**Fig. 5:**
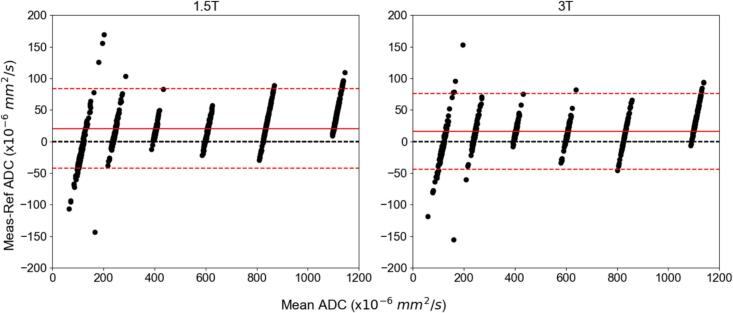
Bland-Altman plot analysis between 1.5T and 3T MRI systems with 95 % limits of agreement represented by dashed red lines and the mean bias represented with a solid red line. The difference was determined by the measured value minus the reference value. (For interpretation of the references to colour in this figure legend, the reader is referred to the web version of this article.)

**Fig. 6:**
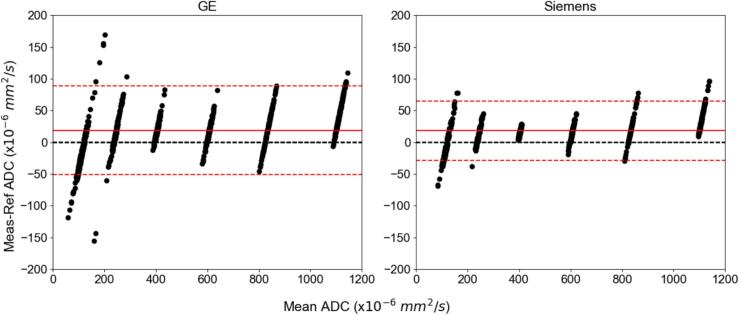
Bland-Altman plot analysis between GE and Siemens MRI systems with 95% limits of agreement represented by dashed red lines and the mean bias represented with a solid red line. The difference was determined by the measured value minus the reference value. (For interpretation of the references to colour in this figure legend, the reader is referred to the web version of this article.)

In Fig. 5, Fig. 6, the results from the Bland-Altman analysis are displayed as stratified by different magnetic field strengths and manufacturers, respectively. A similar visualization across platforms is shown in Supplementary Fig. S2. The 95 % limits of agreement were calculated and overlaid on each plot, as well as the means to represent any overall bias.

## 4 Discussion

To our knowledge, this is the largest study using the NIST/QIBA DWI phantom to characterize diffusion parameters across scanners of various manufacturers, models, and field strengths. We found that most ADC values were slightly overestimated by ∼ 5 % by following the QIBA acquisition protocol and that higher concentrations of PVP, and thus lower ADC, resulted in reduced SNR and higher CoV. Secondly, the lower ADC values also resulted in higher measured error rates compared to NIST-traceable reference values. Thirdly, the sagittal orientation produced a higher CoV across each orientation. Finally, 1.5T systems performed similarly compared to 3T systems while the GE systems tested here produced wider 95 % confidence intervals compared to Siemens upon Bland-Altman analysis.

In more detail, from Fig. 1, the large percent error seen primarily at the 50 % PVP vials or low ADC values was shown to be a consequence of the SNR dependence. The large CoV in Fig. 4 for the vials with the higher PVP concentration also showed them to be the least reliable. As indicated by Fig. 3, the sagittal orientation had higher ADC accuracy across all scanners. This could potentially be due to gradient nonlinearities within each system. The 95 % limits of agreement were similar between 1.5T and 3T acquisitions with measurements falling outside the range at the lower ADC values. Comparing between GE and Siemens MRI platforms, the Siemens’ limits were narrower with less measurements outside the 95 % confidence interval limits. This implies that GE was more likely to deviate from references values more unpredictably compared to the expected scanner population performance, especially in the lower ADC vials. However, only 12 sets of data were acquired on Siemens MRI systems compared to 19 sets on GE systems. Fig. 5, Fig. 6 showed different intervals for their respective limits of agreement. All systems seem to be close to having zero bias on average with a slightly increased error for the highly restricted, low SNR, ADC values and negative bias for high diffusion values. This overestimation in ADC could be due to the inclusion of the b-value of 0 s/mm^2^ which may artificially increase the ADC since it includes potential perfusion effects [31]. Therefore, future studies should investigate slightly higher lowest b-values of around 50–100 s/mm^2^. With the Bland-Altman analysis, it was observed that primarily lower ADC value measurements were outside the 95 % limit. With the known ADC value being small, these differences produced large percent errors, compared to the same differences at the larger ADC values producing small percent errors. The nature of this study called for a consistent method to calculate ADC values across MRI scanners, as done so through QIBAPhan. While this method investigated a robust characterization of ADC values across vendors, models, and field strengths, the ADC values found in this study may vary more in direct radiation oncology applications, as optimized clinical workflows use built-in on-board commercial software for such calculations. Finally, from the ANOVA results, the mean and standard deviation of the percent errors from each phantom, which were used interchangeably on different scanners, were not significantly different from one another. This implies that multiple phantoms can be used to build an ADC quality assurance program in a large institution without fear of significant differences between different versions of the same phantom.

One potential limitation of test fluid and DWI phantom is that their diffusion properties depend on their temperature; previous studies display an increase in ADC value with increasing temperature[32]. To get around this limitation, the NIST/QIBA phantom puts the PVP test fluid in equilibrium in an ice water bath to maintain a constant temperature of 0 °C, which is the approach used in this study. Care has been taken in the preparation and temperature measurement of the phantom prior to scanning to ensure consistent diffusion property. This is complemented by the fact that the temperature dependence is an exponential function and relatively flat at 0 °C in general for the range of PVP percentage and flatter with higher percentage of PVP. Therefore, the diffusion properties of the NIST/QIBA phantom should be relatively stable. Another potential limitation is that we did not consider an SNR-weighted fitting for the ADC calculation which may lead to bias at higher b-values where the SNR is lower [33]. Future work will investigate SNR dependency and compare measurements without the higher 2000 s/mm^2^ b-value. Bridging from this, the recommended QIBA profile [25] includes a lowest b-value of 0 s/mm^2^ which can lead to unintended perfusion effects [31]. A final limitation is the use of EPI as the DWI acquisition strategy which is known to suffer from geometric distortion, thus limiting its application in radiation oncology settings where accuracy is critical, particularly in the head and neck region with large air cavities enhancing this distortion [14]. Future studies should investigate distortion-limiting acquisitions such as RESOLVE (Siemens), BLADE (Siemens), PROPELLOR (GE), and SPLICE (Philips).

This study is an important step towards quantitative DWI implementation in the radiation oncology space. There is an unmet need for standardization of ADC values to accurately delineate tumors from normal tissue across the various MRI systems and clinical applications. For instance, by combining the effects of perfusion MRI with DWI, high b-value diffusion MRI can be utilized to diagnose glioblastoma [34]. Additionally, ADC values can be standardized to specific malignancies, including those in the head and neck [35,36], breast [37], and thoracic regions [38]. The results from this study, particularly the biases seen across varying MRI manufacturers, motivate its replication across each clinic to minimize reproducibility biases in the multi-timepoint settings common in radiation oncology. It may also be used in a retrospective setting to confirm whether multiple ADC measurements can be included in a longitudinal analysis and the associated uncertainties involved. The focus here would be to match systems with similar levels of CoV across slice orientations and ADC values to prioritize consistent measurements compared to an optimal measurement inconsistently to best follow-up the subjects. For example, the “1.5T Siemens Aera (6)” and “3T GE HDxt (2) – whole” scanners demonstrated a similar CoV across each slice orientation which is unexpected due to both cross-field strength and cross-vendor differences and, therefore, could be matched for baseline and follow-up imaging. Prior to implementation, the bias across ADC values should also be compared between the two to ensure repeatable and reproducible measurements. From the results presented here, an argument could be made that the follow-up in the same tissues can be made on either 1.5T or 3T scanners given that the measured spread in measured values is similar between the two field strengths at the tissue’s ADC. However, this should be used with caution as each software version and equipment upgrade (i.e., coils) may result in unexpected changes necessitating re-evaluation. For this reason, updating the results of this study following significant changes onto a dashboard-type display, or similar, may be helpful to monitor clinic operations from a physics point-of-view. It may be debated whether including monthly, weekly, or even daily ADC measurements along with the daily ACR phantom quality assurance measurements would be useful towards achieving these goals. With more data across different MRI platforms, future work would involve creating conversion factors to help facilitate quantitative measurements for multi-platform and multi-site imaging studies. Such conversion factors could be calculated from the percent errors seen across ADC values to adjust each scanner’s output to a synthetic 0 % error, though future studies must address the comparison between bias seen in phantoms compared to human subjects. If successful, the resulting “bias vs. ADC value” curve could be used to correct for human subject measurements for longitudinal assessment and clinical trials. It may be useful for MRI manufacturers to standardize how ADC maps are saved. For example, saving both a noise threshold for image interpretation and non-threshold ADC map for quality assurance would be useful in retrospective studies to prevent re-calculation of ADC maps. Further, the methods for image intensity normalization across the b-values should be clearly specified.

In this study, acquisition and analysis of DWI data from numerous MRI systems from a single-institution characterized ADC accuracy and reliability. With the increasing usage of DWI in the clinic, a quantitative quality assurance process should be implemented to provide improved quality control over the MRI systems which would consistently acquire the data needed to reproduce this study at a daily frequency allowing for a more comprehensive understanding of clinic shortcomings and future opportunities. Through ADC standardization across various vendors, scanners, field strengths, and acquisition parameters, radiation oncology is closer to recognizing ADC as a quantitative imaging biomarker. In characterizing ADC values across 23 different scanners as done in this study, DWI can be utilized in a larger number of clinics internationally with a more reliable performance.

## 5 Funding statement

LM is supported by a National Institutes of Health (NIH) Diversity Supplement (R01CA257814-02S2). NW is supported by a training fellowship from UTHealth Houston Center for Clinical and Translational Sciences T32 Program (Grant No. T32 TR004905) and a NIH National Institute of Dental and Craniofacial Research (10.13039/100000072NIDCR) Academic Industrial Partnership Grant (R01DE028290). CDF received/receives funding and salary support related to this project during the period of study execution from: the National Institutes of Health (NIH) National Institute of Biomedical Imaging and Bioengineering (10.13039/100000070NIBIB) Research Education Programs for Residents and Clinical Fellows Grant (R25EB025787-01); the National Institute for Dental and Craniofacial Research Establishing Outcome Measures Award (1R01DE025248/R56DE025248) and Academic Industrial Partnership Grant (R01DE028290); NCI Early Phase Clinical Trials in Imaging and Image-Guided Interventions Program (1R01CA218148); an NIH/NCI Cancer Center Support Grant (CCSG) Pilot Research Program Award from the UT MD Anderson CCSG Radiation Oncology and Cancer Imaging Program (P30CA016672); an NIH/NCI Head and Neck Specialized Programs of Research Excellence (SPORE) Developmental Research Program Award (P50 CA097007). CDF received/receives funding and salary support unrelated to this project during the period of study execution from: NIH Big Data to Knowledge (BD2K) Program of the National Cancer Institute (NCI) Early Stage Development of Technologies in Biomedical Computing, Informatics, and Big Data Science Award (1R01CA2148250; National Science Foundation (NSF), Division of Mathematical Sciences, Joint NIH/NSF Initiative on Quantitative Approaches to Biomedical Big Data (QuBBD) Grant (NSF 1557679); NSF Division of Civil, Mechanical, and Manufacturing Innovation (10.13039/100000147CMMI) grant (NSF 1933369); and the Sabin Family Foundation. Direct infrastructure support is provided to CDF by the multidisciplinary Stiefel Oropharyngeal Research Fund of the University of Texas MD Anderson Cancer Center Charles and Daneen Stiefel Center for Head and Neck Cancer and the Cancer Center Support Grant (P30CA016672) and the MD Anderson Program in Image-guided Cancer Therapy.

## Declaration of competing interest

The authors declare the following financial interests/personal relationships which may be considered as potential competing interests: LM has received unrelated travel funding from Elekta AB. CDF has received direct industry grant support, honoraria, and travel funding from Elekta AB unrelated to this project. Part of the research was conducted at the Center for Academic Biomedical Imaging at the University of Texas MD Anderson Cancer Center with equipment support from GE Healthcare.
