# Supplementary material for: Quantitative evaluation of apparent diffusion coefficient in a large multi-unit institution
Source: Phys Imaging Radiat Oncol. 2025 Oct 30;36:100856. doi: 10.1016/j.phro.2025.100856 (PMC12637080; doi:10.1016/j.phro.2025.100856)
Supplement: Supplementary Data 1 [file mmc1.docx]

Supplementary Materials


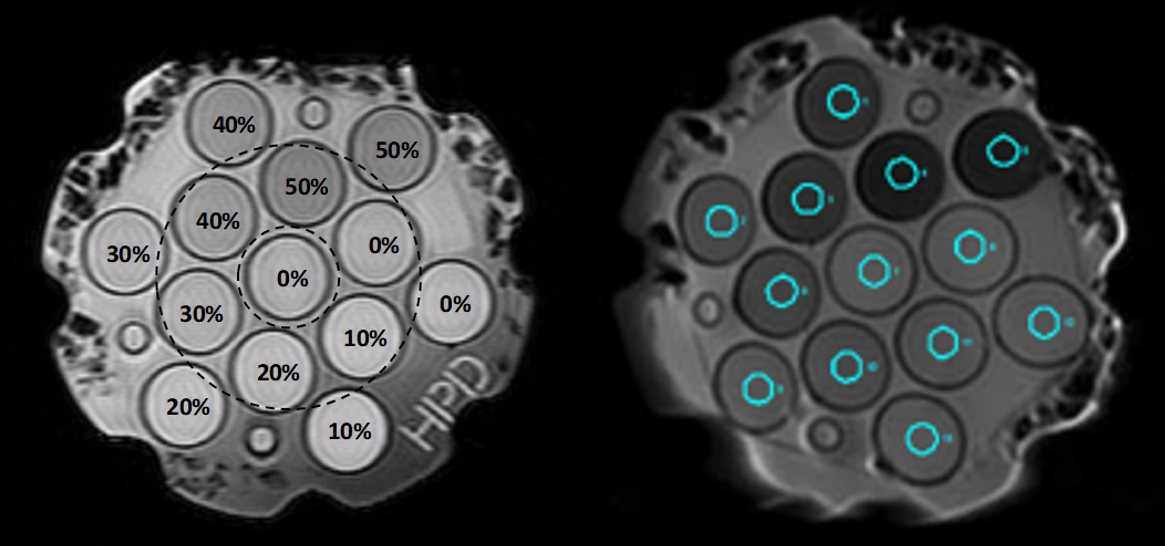


**Figure S1:** Left) A MR image showing the 30 mL vials of aqueous solutions of PVP. Each solution has an inner and outer vial. The inner vials are located immediately adjacent to the center vial (between the dotted lines); and the outer vials are located on the periphery (outside the dotted lines). Right) Typical example of diffusion-weighted image analysis showing the user-selected regions of interest (cyan) overlaid on each of the 13 internal vials. ROIs were sized and placed in a manner to avoid partial volume effects with the vial edge.

**Table S1:** List of MRI systems and their respective performance specifications that were used in the study.

| **Vendor** | **Platform** | **Field Strength (T)** | **Bore Size (cm)** | **Gradient Performance** | **Number of Systems** | **Coil Used** |
| --- | --- | --- | --- | --- | --- | --- |
| Siemens | Aera | 1.5 | 70 | 45 mT/m at 200 T/m/s | 8 | 20-channel head and neck |
|  | Espree | 1.5 | 70 | 33 mT/m at 170 T/m/s | 1 | 20-channel head and neck |
|  | Skyra | 3.0 | 70 | 45 mT/m at 200 T/m/s | 1 | 20-channel head and neck |
|  | Prisma | 3.0 | 70 | 80 mT/m at 200 T/m/s | 1 | 64-channel head and neck |
| GE | HDxt | 1.5 | 60 | 33 mT/m at 120 T/m/s | 5 | 8-channel brain array; 16-channel head, neck, and spine array |
|  |  | 3.0 | 60 | whole: 23 mT/m at 80 T/m/s zoom: 40 mT/m at 150 T/m/s | 2 | 8-channel brain array |
|  | MR450w | 1.5 | 70 | 33 mT/m at 120 T/m/s | 2 | 16-channel GEM head and neck unit |
|  | MR750w | 3.0 | 70 | 33 mT/m at 120 T/m/s | 2 | 16-channel GEM head and neck unit |
|  | MR750 | 3.0 | 60 | 50 mT/m at 200 T/m/s | 1 | 8-channel brain array; 32-channel head |

**Table S2:** ADC values of each vial at 0°C given by the manufacturer. The center vial is located in the center; the inner vials are located immediately adjacent to the center vial; and the outer vials are located on the periphery, as shown in **Supplementary Materials**, **Figure 1**.

| **Concentration (%)** | **Vial location** | **References**  **(x10^-6^ mm^2^/s)** |
| --- | --- | --- |
| 0 | Center | 1091.060571 |
| 0 | Inner | 1090.134463 |
| 0 | Outer | 1090.21889 |
| 10 | Inner | 824.0288105 |
| 10 | Outer | 824.1549136 |
| 20 | Inner | 596.9578014 |
| 20 | Outer | 599.359388 |
| 30 | Inner | 393.699701 |
| 30 | Outer | 396.467332 |
| 40 | Inner | 235.1549486 |
| 40 | Outer | 238.4391252 |
| 50 | Inner | 118.275699 |
| 50 | Outer | 122.9606508 |


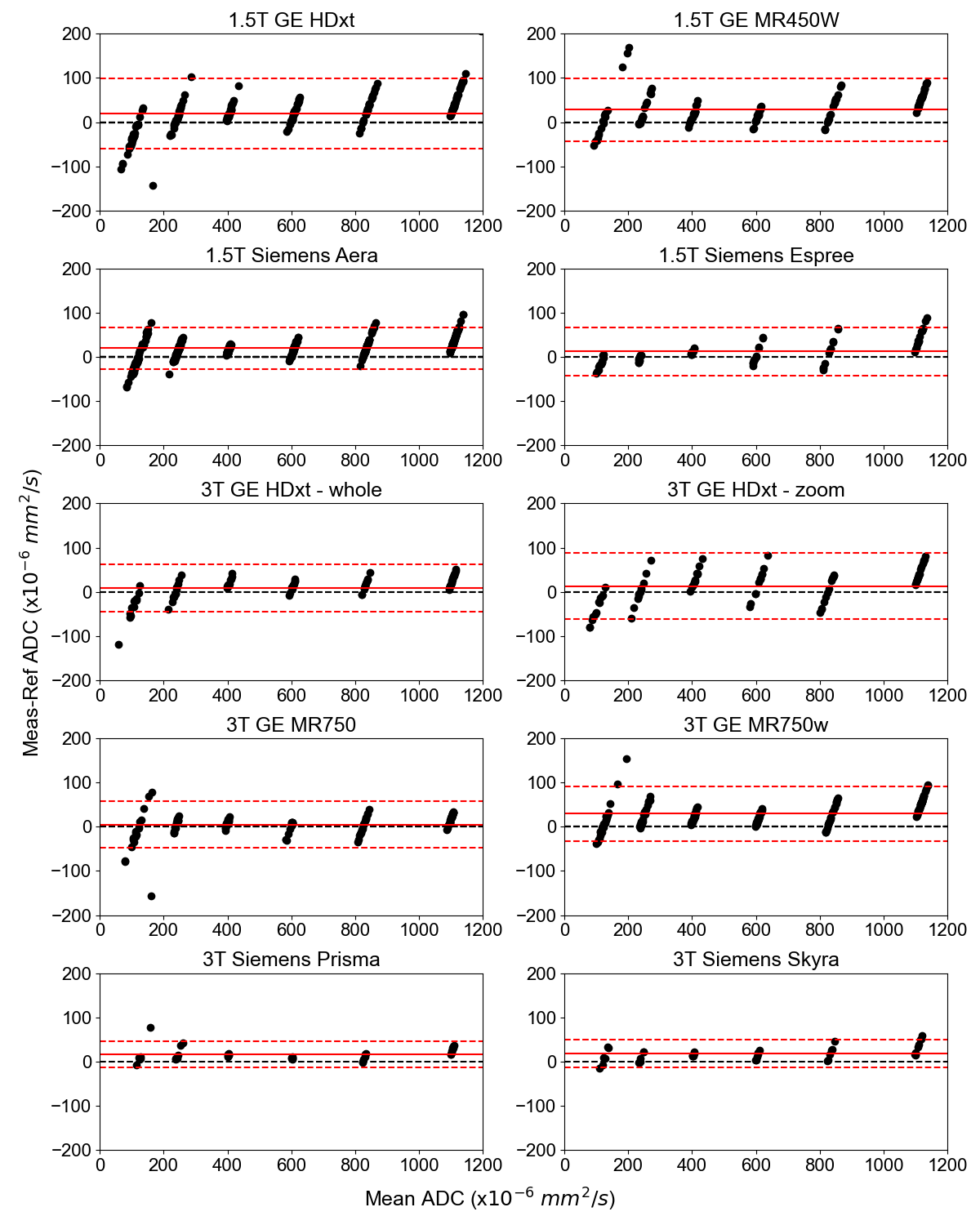


**Figure S2:** Bland-Altman plot analysis between different MR systems with 95% limits of agreement represented by dashed red lines and the mean bias represented with a solid red line. The difference was determined by the measured value minus the reference value.
